# Sporozoite immunization of human volunteers under chemoprophylaxis induces functional antibodies against pre-erythrocytic stages of *Plasmodium falciparum*

**DOI:** 10.1186/1475-2875-13-136

**Published:** 2014-04-05

**Authors:** Marije C Behet, Lander Foquet, Geert-Jan van Gemert, Else M Bijker, Philip Meuleman, Geert Leroux-Roels, Cornelus C Hermsen, Anja Scholzen, Robert W Sauerwein

**Affiliations:** 1 Radboud University Medical Center, Department of Medical Microbiology, Geert Grooteplein 28, Microbiology 268, Nijmegen, HB 6500, The Netherlands; 2 Center for Vaccinology, Ghent University and University Hospital, De Pintelaan 185, Ghent 9000, Belgium

**Keywords:** Malaria, *Plasmodium falciparum*, Sporozoites, Liver-stage, Immunization, CPS, CHMI, Inhibitory antibodies, Human liver-uPA-SCID mouse model

## Abstract

**Background:**

Long-lasting and sterile protective immunity against *Plasmodium falciparum* can be achieved by immunization of malaria-naive human volunteers under chloroquine prophylaxis with sporozoites delivered by mosquito bites (CPS-immunization). Protection is mediated by sporozoite/liver-stage immunity. In this study, the capacity of CPS-induced antibodies to interfere with sporozoite functionality and development was explored.

**Methods:**

IgG was purified from plasma samples obtained before and after CPS-immunization from two separate clinical trials. The functionality of these antibodies was assessed *in vitro* in gliding and human hepatocyte traversal assays, and *in vivo* in a human liver-chimeric mouse model.

**Results:**

Whereas pre-treatment of sporozoites with 2 mg/ml IgG in the majority of the volunteers did not have an effect on *in vitro* sporozoite gliding motility, CPS-induced IgG showed a distinct inhibitory effect in the sporozoite *in vitro* traversal assay. Pre-treatment of *P. falciparum* sporozoites with post-immunization IgG significantly inhibited sporozoite traversal through hepatocytes in 9/9 samples when using 10 and 1 mg/ml IgG, and was dose-dependent, resulting in an average 16% and 37% reduction with 1 mg/ml IgG (p = 0.003) and 10 mg/ml IgG (p = 0.002), respectively. *In vivo*, CPS-induced IgG reduced liver-stage infection and/or development after a mosquito infection in the human liver-chimeric mouse model by 91.05% when comparing 11 mice receiving post-immunization IgG to 11 mice receiving pre-immunization IgG (p = 0.0008).

**Conclusions:**

It is demonstrated for the first time that CPS-immunization induces functional antibodies against *P. falciparum* sporozoites, which are able to reduce parasite-host cell interaction by inhibiting parasite traversal and liver-stage infection. These data highlight the functional contribution of antibody responses to pre-erythrocytic immunity after whole-parasite immunization against *P. falciparum* malaria.

## Background

Malaria is caused by mosquito-transmitted protozoan *Plasmodium falciparum* parasites with a complex multi-stage life cycle in the human host. When *P. falciparum*-infected *Anopheles* mosquitoes probe for blood, sporozoites are deposited in the skin, move by circular locomotion (gliding) [1], and traverse cell barriers by breaching host cell membranes [2]. When sporozoites have reached the liver via the blood circulation, they first cross the sinusoidal barrier, traverse through and eventually invade hepatocytes [2,3]. Previous studies have demonstrated the importance of *P. falciparum* sporozoite gliding motility for invasion of a hepatocyte [4,5]. Moreover, cell traversal has been shown to be important for the progression of sporozoites to the liver and thus, enhancement of successful infection [3,6-8]. Subsequent liver-stage development is completed by merozoite release into the bloodstream and invasion of erythrocytes (asexual blood-stages) [9].

Immunity against malaria can be naturally acquired in individuals living in malaria-endemic areas, however, only after continuous exposure to the parasite, and appears to wane in the absence of ongoing *P. falciparum* exposure [10]. Historic passive transfer studies have demonstrated a key role for antibodies in controlling blood-stage parasites during natural *P. falciparum* infection and reducing clinical symptoms of malaria [11-13], as confirmed in animal models of malaria [14-16]. Although antibodies are crucial in controlling blood-stages, naturally acquired immunity never results in complete parasite elimination.

Generating long-lasting and sterilizing immunity against malaria with pre-erythrocytic subunit vaccines, has only had limited success. The RTS,S subunit vaccine, to date the only most advanced malaria vaccine candidate tested in Phase III clinical trials, is based on *P. falciparum* circumsporozoite protein (CSP), a major sporozoite surface protein [17]. The RTS,S vaccine has been shown to elicit strong CSP-specific antibodies and to induce protection in the majority of the volunteers in a CHMI model upon infectious mosquito bite challenge [18-21]. However, 4/5 and 5/9 protected volunteers developed delayed parasitaemia upon re-challenge with infectious mosquito bites ~ six or five months after the initial challenge, respectively [21,22]. Additionally, RTS,S vaccination only confers modest protection in the field [23-26]. Immunization with other subunit vaccines, for instance with the sporozoite surface protein 2 (a homolog for *P. falciparum* thrombospondin-related adhesion protein (TRAP)) which is expressed on both the surface of sporozoites [27,28] and within infected hepatocytes [28], induced only partial protection in mice, but complete protection when given together with CSP [29]. However, Phase I/IIa clinical trials in which humans were immunized with RTS, S/TRAP failed to provide protection in the majority of volunteers (unpublished data, as written by [30]). Moreover, immunization of humans with a recombinant liver-stage antigen-1 (LSA-1)-based vaccine elicited high antibody titers, but did also not protect against *P. falciparum* infection [31].

While the induction of sterile immunity with subunit vaccines has been shown to be difficult, sterile immunity against malaria can be achieved experimentally by whole-parasite immunization with attenuated sporozoites in animal models and human volunteers, targeting the sporozoite/liver-stage parasites (pre-erythrocytic stages). As early as the 1960s, it was shown that sterile protective immunity can be induced in animals and humans by immunization with *Plasmodium* sporozoites attenuated by gamma irradiation (RAS) and delivered by *P. falciparum*-infected mosquito bites [32-34]. Sera from RAS-immunized subjects were able to reduce sporozoite invasion into hepatocytes, as shown in both animal [35,36] and human models of malaria [37-39]. This immunization approach, however, requires a minimum of 1,000 bites from irradiated mosquitoes to induce sterilizing immunity in humans. Therefore, more studies to other potential immunization methods have been conducted. Very recently, it has been demonstrated that intravenous immunization of humans with a radiation-attenuated and cryopreserved *P. falciparum* sporozoite vaccine can also induce protection against *P. falciparum* malaria [40]. *In vitro* invasion experiments with hepatoma cells and sera from volunteers protected after 4–5 immunization doses revealed that immunization-induced antibodies were able to inhibit *in vitro* sporozoite invasion [40]. However, to circumvent the need for irradiation of parasites, another whole-parasite vaccination approach has been developed, to be specific the genetically engineered and attenuated parasites (GAP). Passive immunization of naive mice with pooled serum from mice immunized i.v. with *Plasmodium yoelii*-GAP one day before mosquito bite challenge, reduced the liver parasite burden by 48 hours after infection [41]. Exposure of human volunteers to ~5 bites from mosquitoes infected with a first generation of *P. falciparum* GAP (*Pf*GAP), followed by a high-dose exposure to ~200 *Pf*GAP-infected mosquito bites, led to breakthrough infection in one out of six volunteers, consisting of breakthrough *Pf*GAP-parasites [42]. While this Phase I study was not safe and therefore not successful, plasma from volunteers obtained three months after high-dose exposure could efficiently block *in vitro* hepatoma cell invasion by *P. falciparum* sporozoites [43].

Next to the RAS and GAP whole-parasite vaccination approaches, a highly efficient immunization regimen based on controlled human malaria infection (CHMI) that induces long-lasting sterile immunity in malaria-naive individuals has recently been established: volunteers are exposed to ~45 wild type *P. falciparum*-infected mosquito bites, while receiving a prophylactic regimen of chloroquine (ChemoProphylaxis and Sporozoites, CPS) [44,45]. CPS-immunization allows full liver-stage maturation and development of early asexual blood-stage parasites, however, it induces protection specifically targeting pre-erythrocytic, but not blood-stages [46]. In previous CPS-immunization studies, cellular responses to both blood-stage parasites and sporozoites were found [45,47]. Antibodies targeting the cell-free sporozoites may interfere with migration and invasion of hepatocytes, and thus lower the initial liver parasite load and complement T-cell mediated protection. Analysis of antibody responses by *P. falciparum* protein-microarray identified two pre-erythrocytic antigens i.e. CSP and LSA-1 as the two proteins predominantly recognized after CPS-immunization [48]. Moreover, efficient induction of memory B-cells and antibodies was found to classical pre-erythrocytic antigens, to be specific CSP and LSA-1 (Nahrendorf and Scholzen *et al*., manuscript in preparation). While the induction of antibodies by CPS-immunization has been demonstrated, their functional activity against sporozoite/liver-stage parasites has not yet been established. This study, therefore, focused on the possible functional contribution of antibodies to pre-erythrocytic protective immunity after CPS-immunization.

## Methods

### Study design and plasma sample collection

Citrated plasma samples were collected during two clinical CPS-immunization trials approved by the Central Committee for Research Involving Human Subjects of The Netherlands (Study 1 [44], ClinicalTrials.gov number NCT00442377; CCMO NL24193.091.09, and Study 2 [46], ClinicalTrials.gov number NCT01236612; CCMO NL34273.091.10). In both studies, healthy malaria-naive Dutch volunteers were immunized three times at monthly intervals by exposure to 12–15 *P. falciparum*-infected mosquito bites, while receiving chloroquine prophylaxis. All subjects provided written informed consent before screening and the study team complied with the Declaration of Helsinki and Good Clinical Practice. The following plasma samples, collected before (pre) and after CPS-immunization (post), were selected for IgG purification based on availability: ten volunteers from Study 1 and three from Study 2 protected against sporozoite challenge infection and another three volunteers from Study 2 challenged with blood-stages. For the latter three, their protection status upon sporozoite challenge is therefore unknown.

### IgG purification from citrated plasma samples

IgG purification from 3–8 ml plasma samples was performed using a 5 ml HiTrap Protein G HP column (Amersham Biosciences) according to manufacturer’s instructions and IgG was taken up in phosphate buffered saline (PBS, GIBCO). IgG concentrations were determined by a NanoDrop spectrophotometer (NanoDrop Technologies, NanoDrop program 1000 version 3.8.2.). For Study 1, due to limited plasma availability, IgG samples from two volunteers each were pooled, resulting in five pools of two volunteers. For Study 2, IgG samples from six individual CPS-immunized volunteers were available.

### Generation of *P. falciparum* sporozoites

*Plasmodium falciparum* NF54 asexual and sexual blood stages were cultured in a semi-automated culture system [49-51]. Sporozoites were produced by feeding female *Anopheles stephensi* mosquitoes using standard membrane feeding of cultured gametocytes [52]. For *in vitro* assays, salivary glands were hand dissected, collected in Leibovitz culture medium (Sigma Aldrich) supplemented with 10% foetal bovine serum (FBS, GIBCO), homogenized in a homemade glass grinder. Sporozoites were counted in a Bürker-Türk counting chamber using phase-contrast microscopy.

### Gliding assay

Gliding assays were performed in eight-chamber glass Lab-Tek chamber slides (Nalgene, Nunc) pre-coated with 25 μg/ml monoclonal anti-CSP (3SP2, [53]) to capture shed *P. falciparum* circumsporozoite protein. Sporozoites were pre-incubated in duplicate with 2 mg/ml pre- or post-immunization IgG (due to limited plasma/IgG availability) in the presence of 10% FBS for 30 min on ice and then sporozoite/IgG suspension (30,000 spz/well) was added to each well. After 90 min of incubation at 37°C in 5% CO_2_, gliding trails were fixed with 4% paraformaldehyde (PFA) for 20 min at room temperature (RT). Each well was washed twice with PBS, blocked with 10% FBS in PBS for 20 min at RT and washed again. Gliding trails were visualized with 10 μg/ml anti-CSP-FITC in 10% FBS in PBS, incubated for 60 min in the dark at RT, washed and mounted with Fluoromount-G (Southern Biotech) and 24×50 mm cover glasses (VWR International). The number of gliding trails was counted per 280 fields of view (per well) at an enlargement of 1,000× with oil immersion.

### Traversal assay

The functional capacity of CPS-induced antibodies to inhibit sporozoite traversal through human hepatocytes *in vitro* was studied using an optimized flow cytometry based version (Behet *et al*., manuscript in preparation) of the dextran incorporation assay [2,54]. Only cells traversed by sporozoites and, therefore, wounded, incorporate dextran and the percent of dextran-positive cells can be quantified with flow cytometry.

The HC-04 hepatocyte cell line (Homo sapiens HC-04, MRA-975, deposited by Jetsumon Sattabongkot [55]) was obtained through the MR4 as part of the BEI Resources Repository (NIAID, NIH). Cells were cultured in HC-04 cell culture medium, containing equal volumes of F-12 Nutrient Mixture (GIBCO) and Minimal Essential Medium (GIBCO) supplemented with 10% FBS (GIBCO) and 1% penicillin/streptomycin (GIBCO), at 37°C in an atmosphere of 5% CO_2_.

*Plasmodium falciparum* sporozoites were pre-incubated with 10 mg/ml or 1 mg/ml IgG for 30 min on ice. Data from 2 pre-immunization IgG samples from Study 1 were lost for technical reasons. Sporozoites pre-treated with 1.25 μg/ml of the mycotoxin cytochalasin D [56] served as a positive control resulting in a mean traversal inhibition of 93.1% and non-infected cells incubated with dextran served as a background control.

Sporozoites (5.10^4^) were added to 96-well plates containing monolayers of 5.10^4^ HC-04 hepatocytes in the presence of 0.5 mg/ml fixable tetramethylrhodamine dextran (10,000 MW, D1817, Invitrogen) in duplicates or triplicates, centrifuged at 3,000 RPM for 10 min at RT with a low brake (Eppendorf Centrifuge 5810 R) and incubated for 2 h at 37°C in 5% CO_2_. After incubation, wells were gently washed three times with PBS to remove extracellular dextran, trypsinized with 0.05% Trypsin-EDTA (GIBCO) for 5 min at RT, taken up in 10% FBS in PBS, and centrifuged at 3,600 RPM for 5 min at RT (Eppendorf Centrifuge 5415 D). Cells were re-suspended in 1% PFA in PBS and stored at 4°C in the dark until flow cytometric analysis on an ADP Cyan flow cytometer (Beckman Coulter). Sporozoite traversal was corrected for background dextran incorporation and the percent inhibition of traversal was calculated as follows: 1 – (average% dextran-positive cells in post-immunization IgG cultures/average% dextran-positive cells in pre-immunization IgG cultures) × 100%.

### Passive immunization of human liver-chimeric mice and sporozoite challenge

Human liver-chimeric mice over-expressing urokinase-type plasminogen activator on a severe combined immunodeficiency background (uPA^+/+^-SCID mice) were generated as previously described [57,58]. Cryopreserved primary human hepatocytes (±1.10^6^ cells/mouse, all from the same donor and purchased from BD Gentest (Erembodegem, Belgium) were injected in the spleens of uPA^+/+^-SCID mice within two weeks after birth [57]. Human albumin levels were measured at different time points after transplantation by using the Human Albumin ELISA Quantitation kit (Bethyl Laboratories Inc., Montgomery, TX). Mice with human albumin levels of >2 mg/ml were considered successfully engrafted and used for infection studies. All procedures were approved by the Animal Ethics Committee of the Faculty of Medicine and Health Sciences of the Ghent University (ECD 11/03).

The day before *P. falciparum* infection by mosquito bites, human liver-chimeric mice were injected intraperitoneally with 10 mg of post-immunization IgG from five sets of two CPS-immunized volunteers per pool (Study 1) and six CPS-immunized volunteers (Study 2). Each mouse was injected with one IgG sample, and mice injected with pre-immunization IgG served as controls. Mice were challenged by exposure to *P. falciparum*-infected mosquito bites as previously described [58]. Briefly, the abdomen and chest of mice were shaved with electric clippers and subsequently, mice were positioned on a cardboard box containing 20 *P. falciparum*-infected mosquitoes (one mouse per box) and exposed for 20 minutes to infectious mosquito bites. Successful blood feeding (median: 16 mosquitoes) and sporozoite presence (100%) was confirmed by mosquito dissection after the challenge experiment [59,60].

Due to limited availability of liver-chimeric mice, each IgG sample was injected in one mouse. The relatively wide range in liver load in control mice receiving only infectious mosquito bites as described previously [58,61], was also reflected in the present study in the group of mice receiving pre-immunization IgG. This precludes paired analysis of mice receiving pre- and post-immunization IgG from the same donor, and only allows to compare the pre- and post-immunization groups at group level.

### Isolation of DNA and quantification of *P. falciparum* and human hepatocyte DNA

Isolation of liver DNA and quantification of *P. falciparum* and human hepatocyte DNA was performed as previously described [58]. Briefly, five days after sporozoite challenge mice were maximally bled and sacrificed by cervical dislocation. The removed livers were cut into 12 standardized sections and stored in RNALater (Ambion) at 4°C until analysis. For DNA extraction, 25 mg (±0.1 mg) liver tissue was taken from each section and *P. falciparum*, mouse and human hepatocyte DNA levels were quantified in a total of 300 mg of liver tissue (12 sections × 25 mg; ~25% of total liver) using a highly sensitive qPCR assay, targeting *Pf*18SRNA and mouse and human prostaglandin E receptor (PTGER2) genes, respectively [58,62]. The quantification of the relative amount of human and mouse hepatocytes in mixed liver tissues allowed us to assess the repopulation of chimeric mouse livers with human hepatocytes, and thus to express *P. falciparum* liver infection as a number of parasites per 10^6^ human hepatocytes [58,63]. DNA extracts from titrated samples of ring-stage *P. falciparum*-infected erythrocytes that were spiked with extracted DNA from a uninfected humanized liver, were used for preparation of *P. falciparum* standard curves. Standard curves were prepared by DNA extraction from a titration of defined numbers of human peripheral mononuclear cells (PBMCs) and mouse splenocytes. Percentage calculation was verified by making various ratios of mouse and human DNA extracts [58].

### Statistical analysis

Statistical analysis was performed using GraphPad Prism software version 5 (GraphPad Software Inc., California, USA). For analysis of data of traversal experiments, differences between the percentage of dextran-positive cells in pre- and post-immunization samples were tested using the paired Student’s t-test. Statistical analysis of humanized mouse data was performed using the non-paired, non-parametric Mann Whitney U-test. A p-value of <0.05 was considered significant.

## Results

### Sporozoite gliding motility is not consistently reduced by low IgG concentrations of CPS-induced antibodies

**Figure 1:**
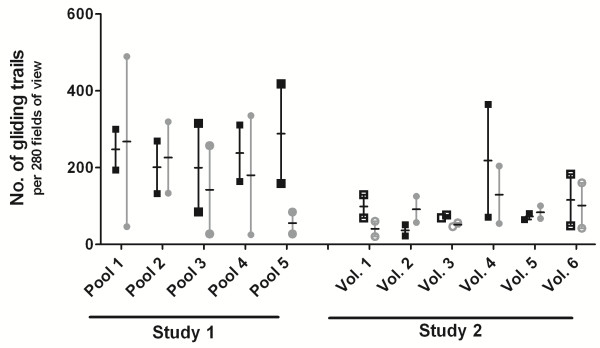
**Effect of CPS**-**induced IgG on *in vitro *sporozoite gliding motility.** The number of sporozoite gliding trails produced by sporozoites incubated with 2 mg/ml IgG was counted in 280 fields per well at an enlargement of 1,000× with oil immersion. Incubations were performed in duplicates and data are expressed as median with range. Pre- or post-immunization IgG samples from five pools of two volunteers each (Study 1) or from six volunteers (Study 2), immunized by ChemoProphylaxis and Sporozoites immunization (CPS), were tested in 3 independent experiments in duplicate. Black squares and grey circles represent the number of gliding trails produced by sporozoites incubated with pre- or post-immunization IgG, respectively. Filled symbols show volunteers protected against mosquito challenge. Open symbols represent volunteers with unknown protection status regarding mosquito challenge (blood-stage challenged).

To explore the capacity of CPS-induced antibodies to interfere with *in vitro* sporozoite gliding motility, sporozoite gliding trails produced by *P. falciparum* sporozoites pre-incubated with 2 mg/ml of either pre- or post-immunization IgG from five pools of two volunteers each (Study 1) or individual IgG samples from six CPS-immunized volunteers (Study 2), were counted by microscopy. *In vitro* sporozoite gliding motility tested was highly variable, not only between volunteers, but also between replicates (Figure 1). In the presence of 2 mg/ml post-immunization IgG, the number of gliding trails was reduced in 3 out of 11 samples (n = 1 from Study 1 (Pool 5), and n = 2 from Study 2 (Volunteers 1 and 3); Figure 1), but in the majority of the volunteers no effect could be observed.

### Sporozoite traversal is dose-dependently inhibited by CPS-induced antibodies

**Figure 2:**
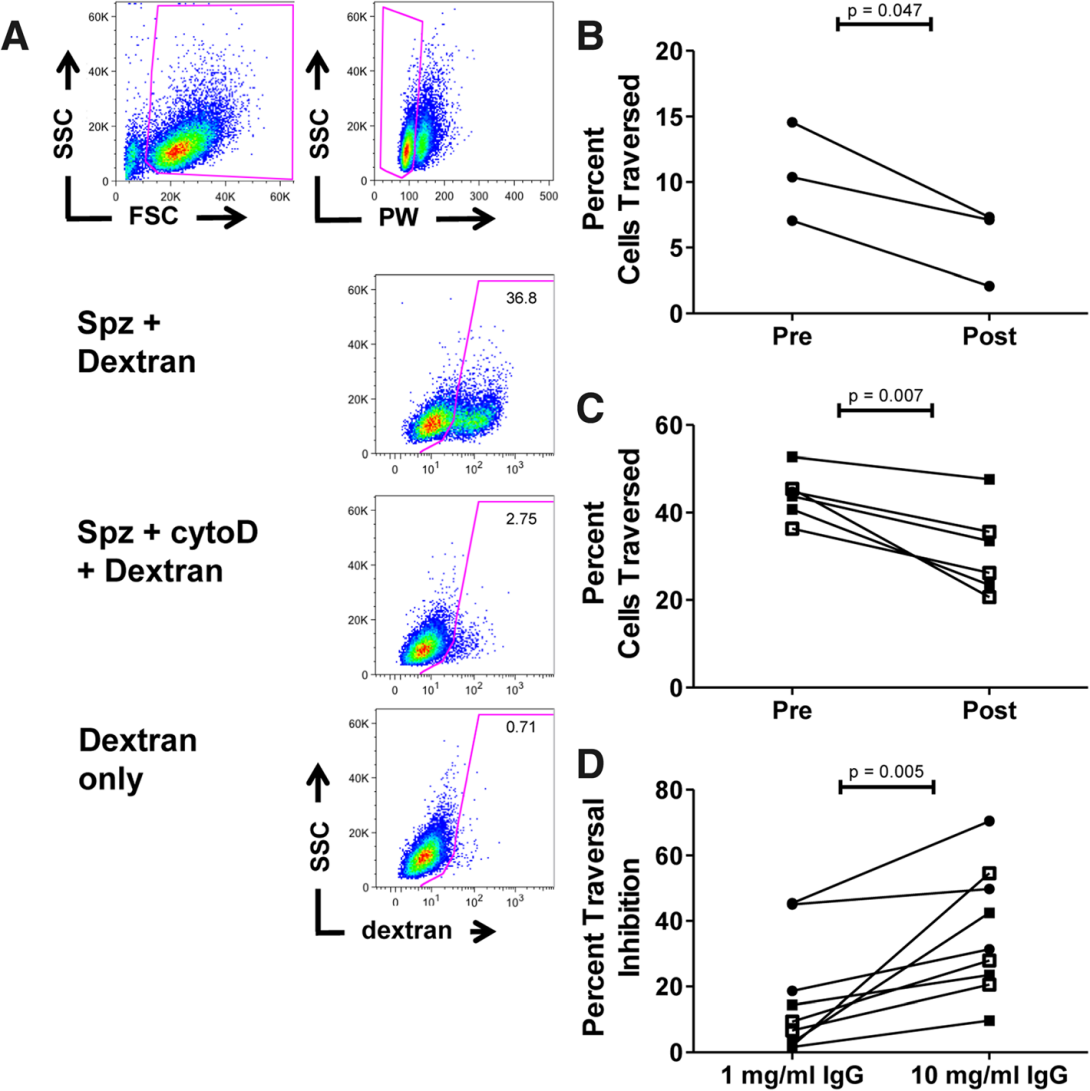
**Effect of CPS**-**induced IgG on *in vitro *hepatocyte traversal. (A)** The proportion of traversed (wounded) cells was determined by flow cytometric analysis, after gating out debris and doublets. Sporozoites with dextran only were used as a positive control and sporozoites pre-treated with cytochalasin D (cytoD) and dextran, as a negative control. Cells incubated with dextran only were used to correct for background. **(B)** Sporozoites were pre-incubated with 10 mg/ml pre- or post-immunization IgG from three pools of two volunteers each (Study 1) or **(C)** from six individual volunteers (Study 2), all of which underwent ChemoProphylaxis and Sporozoites immunization (CPS). Samples were tested in 4 experiments with 3 mosquito batches, in duplicate or triplicates. **(D)** The percentage inhibition of traversal was calculated for post- compared to pre-immunization IgG for each pool/volunteer at a concentration of either 1 or 10 mg/ml. Data are shown as the mean of duplicate or triplicate measurements, and presented as circles or squares for Study 1 and 2, respectively. Filled symbols show volunteers protected against mosquito challenge. Open symbols represent volunteers with unknown protection status regarding mosquito challenge (blood-stage challenged). Differences between pre- and post-immunization IgG, and 1 and 10 mg/ml IgG was tested using the paired Student’s t-test.

Subsequently, the inhibitory activity of immunization-induced antibodies on *in vitro* hepatocyte traversal by sporozoites was investigated. The percentage cells traversed by sporozoites pre-incubated with two concentrations (1 or 10 mg/ml) of either pre- or post-immunization IgG from three pools of two CPS-immunized volunteers each (Study 1) and six individual CPS-immunized volunteers (Study 2), was determined by flow cytometry (Figure 2A and Additional file 1A and B). Pre-treatment of sporozoites with 10 mg/ml post-immunization IgG from both Study 1 and Study 2 lead to a statistically significant reduction in *in vitro* hepatocyte traversal for all volunteers, compared to pre-immunization IgG treatment (Figure 2B and C; Study 1, p = 0.047; Study 2, p = 0.007). The mean percent traversal inhibition by 10 mg/ml IgG was 36.7% (p = 0.002; 50.5% (range 31.4-70.5%) and 29.8% (range 9.7-54.6%) for Study 1 and 2, respectively). Traversal inhibition was concentration-dependent, and significantly higher for 10 mg/ml compared to 1 mg/ml IgG (p = 0.005; Figure 2D). Nevertheless, even as little as 1 mg/ml post-immunization IgG resulted in a significant inhibition of sporozoite traversal (p = 0.003) of 16.2% (mean; range 1.6-45.5%; Figure 1D). Although the percentage of traversed cells varied between experiments with individual mosquito and sporozoite batches (Figure 2B and C), the variability between duplicate/triplicate measurements within each experiment for each IgG sample was very low (Additional file 1A and B; 1 mg/ml IgG and 10 mg/ml IgG, respectively).

### Functional CPS-induced antibodies inhibit *in vivo* sporozoite infection of the liver

Finally, it was investigated whether *in vitro* inhibitory activity of CPS-induced IgG on sporozoite traversal would translate into inhibition of sporozoite infection of and/or development in the liver *in vivo*, using human liver-chimeric mice infected with *P. falciparum* sporozoites by mosquito bites. In two independent experiments, mice were passively immunized with 10 mg of either pre- or post-immunization IgG from five pools of two volunteers each (Study 1) and six individual CPS-immunized volunteers (Study 2) the day before challenge infection. qPCR analysis was performed on liver sections from all mice to determine the liver parasite burden after five days to allow full liver-stage development of *P. falciparum* parasites. The two groups of mice receiving pre- or post-immunization IgG had comparable levels of repopulation with human hepatocytes and body weight, and there was no statistically significant difference in the number of mosquitoes fed on both groups of mice (Table 1), nor between the two experiments conducted.

**Table 1:** **Reduction of liver parasite burden by CPS**-**induced antibodies**

	**Mouse ID**	**IgG sample ID**	**Time point**	**Human albumin levels (mg/ml)**	**Proportion of HuHEP**^**b **^**in mouse liver (%)**	**Weight mice (g)**	**Number of fed mosquitoes (out of 20)**	**Liver parasite burden (No. *Pf/ *** **10^6 HuHEP)**	**No. of *Pf ***^**c **^**positive liver sections (out of 12)**
**Pre**-**CPS**^**a **^**IgG**									
Study 1	B730R	Pool 1	Pre	2,4	26,0	15,9	14	326,5	10
	K1596RL	Pool 2	Pre	2,6	37,2	11,5	19	209,3	5
	B749RL	Pool 3	Pre	3,0	39,7	12,1	17	77,2	5
	K1589R	Pool 4	Pre	4,0	33,0	12,5	14	280,1	11
	K1576	Pool 5	Pre	3,1	35,0	12,2	20	373,0	10
***Median***				***3,*** ***0***	***35,*** ***0***	***12,*** ***2***	***17***	***280,*** ***1***	***10***
Study 2	K1630LR	Vol.^d^ 1	Pre	3,0	40,1	15,1	16	354,5	7
	B934R	Vol. 2	Pre	2,2	29,1	11,7	18	329,8	4
	B934LR	Vol. 3	Pre	2,6	37,7	12,8	17	37,6	3
	B934L	Vol. 4	Pre	1,7	28,5	9,6	12	463,1	4
	K1575	Vol. 5	Pre	1,8	34,4	17,3	15	342,1	2
	B881R	Vol. 7	Pre	2,2	33,0	8,4	13	300,3	8
***Median***				***2,*** ***2***	***33,*** ***7***	***12,*** ***3***	***16***	***336,*** ***0***	***4***
***Median Pre-CPS IgG Study 1 and 2:***				***2,*** ***6***	***34,*** ***4***	***12,*** ***2***	***16***	***326,*** ***5***	***5***
**Post**-**CPS IgG**									
Study 1	K1577L	Pool 1	Post	1,9	29,2	15	16	90,5	1
	K1540L	Pool 2	Post	2,6	31,6	12,6	18	13,1	3
	B733	Pool 3	Post	5,2	40,8	6,7	16	46,4	2
	K1589	Pool 4	Post	4,5	35,9	13,1	15	20,8	3
	B750L	Pool 5	Post	2,5	33,4	12,2	16	302,6	6
***Median***				***2,*** ***6***	***33,*** ***4***	***12,*** ***6***	***16***	***46,*** ***4***	***3***
Study 2	K1666L	Vol. 1	Post	4,6	41,0	15,7	14	37,1	3
	B934	Vol. 2	Post	1,8	17,4	11,7	16	6,7	1
	B905	Vol. 3	Post	4,2	41,2	12,7	18	28,3	2
	K1630L	Vol. 4	Post	1,7	21,2	14,6	14	60,9	2
	B865	Vol. 5	Post	2,3	36,5	17,3	14	29,2	3
	K1683	Vol. 7	Post	2,3	31,3	9,7	15	19,3	3
***Median***				***2,*** ***3***	***33,*** ***9***	***13,*** ***7***	***15***	***28,*** ***8***	***3***
***Median Post***-***CPS IgG Study 1 and 2:***				***2,*** ***5***	***33,*** ***4***	***12,*** ***7***	***16***	***29,*** ***2***	***3***
*Significant difference Post*- *versus Pre*-*CPS IgG*^e^				ns	ns	ns	ns	p = 0.0008	p = 0.003

^a^CPS ChemoProphylaxis and Sporozoites immunization.

^b^HuHEP human hepatocytes.

^c^*Pf P. falciparum.*

^d^Vol. volunteer.

^e^Statistics: significant difference between Post- and Pre-CPS IgG calculated using the non-parametric Mann–Whitney U-test.

Passive immunization of liver humanized uPA^+/+^-SCID mice with post-immunization IgG one day prior to mosquito bite challenge significantly reduced the number of infected hepatocytes by day 5 of infection (Table 1). The median number of liver sections positive for *P. falciparum* infection (*Pf*+) was significantly higher for mice injected with pre- (median 5 *Pf*+ liver sections, interquartile range (IQR) 4–10) than with post-immunization IgG (median 3 *Pf*+ liver sections, IQR 2–3; p = 0.003) and did not statistically differ between Study 1 and 2 samples. Additionally, the median number of *P. falciparum* parasites per million human hepatocytes in mice injected with either pre- or post-immunization IgG was 326.5 (IQR: 209.3- 354.5) and 29.2 (IQR: 19.30-60.90), respectively and did not differ between Study 1 and 2 samples. The reduction of the median liver load by post-immunization IgG was 91.05%, comparing the two groups of mice (n = 11 each) receiving post-immunization or pre-immunization IgG (p = 0.0008).

## Discussion

This study demonstrates for the first time that antibodies with inhibitory activity against pre-erythrocytic stages of *P. falciparum* are induced in human volunteers that have undergone CPS-immunization.

The most striking result was that CPS-induced antibodies could strongly reduce the liver parasite load in human liver-chimeric mice, measured five days after infection by mosquito bite with *P. falciparum* sporozoites. This strong reduction in late stage liver infection (91.05%) might be explained by CPS-induced antibodies targeting sporozoites either in the skin, in the circulation, or during liver infection and/or development. In rodent malaria models, it has been demonstrated that mice immunized by gamma-irradiated *Plasmodium berghei*-infected mosquito bites exhibited high levels of anti-sporozoite antibodies that immobilize sporozoites in the skin by inhibiting their gliding motility and ability to invade dermal blood vessels [64-66]. Therefore, CPS-induced antibodies might also limit the numbers of sporozoites injected into passively immunized mice and immobilizing the sporozoites in the mouse skin, thereby preventing their journey to the liver in the first place. However, the prolonged presence or trapping of sporozoites in the skin after mosquito bites has only been demonstrated in animal models of malaria and not in humans; this may be different for humans. To assess the effect of CPS-induced antibodies on sporozoite ejection during mosquito salivation and sporozoite motility in the skin, *in vitro* mosquito salivation assays [66] and *in vivo* intravital microscopy with Green-Fluorescent Protein-expressing *P. falciparum* parasites [64] can be used, respectively.

The overall liver parasite burden of the control mice (receiving pre-immunization IgG) was lower compared to previous studies [58,61], likely due to mosquito and sporozoite batch variations. Another likely source of variation is the number of injected sporozoites between mice. Nevertheless, the obtained data were consistent across two independent experiments, and the data between two groups of mice receiving either pre- or post-immunization IgG were comparable. Finally, while mice injected with PBS could not be directly included in the IgG experiments due to limited availability of chimeric mouse numbers, PBS-injected mice used in other parallel experiments performed in the same time frame showed the same range in liver parasite burden as mice receiving pre-immunization IgG (Additional file 2).

*In vitro* sporozoite gliding was inhibited by post-immunization IgG in only 3/11 volunteers. This low frequency may be due to the low concentration of IgG that had to be used because of limited supply (2 mg/ml, i.e. 2–5 fold lower than the typical concentration in human plasma). The high intra- and inter-assay variability, despite the fact that always the same operator was carrying out the experiments, may also have contributed to this outcome, which is possibly due to variation between sporozoite batches or methodological factors (e.g. the lack of a centrifugation step to settle sporozoites prior to gliding). Moreover, the manual readout by microscopy assessing shape or length of gliding trails remains somewhat subjective [67]. Overall, this assay appears more suitable for qualitative rather than quantitative analysis, e.g. for studying the phenotype of knock-out parasites [68,69].

The *in vitro* traversal assay, in contrast, is a high-throughput assay and the variation between replicate measurements is very tight (Additional file 1A and B). Although the percentage cells traversed varied between experiments (probably due to variation between sporozoite batches), incubation of sporozoites with 10 μg/ml of the anti-CSP monoclonal antibody (3SP2) consistently results in the same proportional traversal inhibition (median 86.4%; IQR 85.0-89.7%), independent of absolute traversal rates (Additional file 3). Moreover, the variability between duplicate/triplicate measurements for each IgG sample was very low. In contrast to the gliding assay, significant and consistent inhibition of traversal activity of sporozoites by CPS-induced antibodies was found, by as little as 1 mg/ml post-immunization IgG. For three of the volunteers whose IgG was assessed in this study, the protection status against mosquito-bite challenge remains unknown since they received a blood-stage challenge, but they did undergo the same immunization regimen as protected volunteers [46]. Consistent with this, their ability to reduce sporozoite traversal was comparable to that of volunteers known to be protected from mosquito challenge, and 2/3 blood-stage challenged volunteers also showed inhibition of gliding motility.

A dose-dependency for the traversal inhibitory effect of CPS-induced IgG was observed, since traversal inhibition by 10 mg/ml IgG was about 2.2 fold higher than for 1 mg/ml. Traversal inhibition showed a broad range across all volunteers. Possible reasons for this may be the intra-individual differences in antibody specificity, affinity maturation and/or binding capacity to sporozoites. Of note, inhibition of sporozoite traversal *in vitro* did not reach 100 percent, when using an average plasma concentration of 10 mg/ml IgG, and was lower than the inhibition of liver-stage infection and/or development *in vivo*. This might be due to the fact that antibodies do not only interfere with sporozoite hepatocyte traversal, but also with other mechanisms of sporozoite functionality. Another targeted pathway might be hepatocyte invasion, as shown for plasma from RAS- or CPS-immunized mice [36], and for serum, IgG, or monoclonal antibodies derived from human volunteers immunized by RAS [37-39], an irradiated and cryopreserved sporozoite vaccine [40], GAP ([43], or subunit vaccines [61,70] - although it is not clear whether this process can be affected completely independently from traversal, which is a requirement for invasion [71]. Additionally, CPS-induced antibodies might also interfere with intrahepatic parasite development by inducing clearance of infected hepatocytes by means of antibody-dependent cell-mediated cytotoxicity, as shown *in vitro* in murine hepatic cells incubated with a monoclonal antibody recognizing *Plasmodium* heat shock protein 70 [72,73]. SCID mice, while devoid of adaptive lymphocytes, still have an innate immune system including natural killer (NK) cells and macrophages [74,75], both of which are capable of mediating antibody-dependent killing [76]. Whether such a mechanism may also play a role in the *in vivo* liver-chimeric uPA^+/+^-SCID model remains to be investigated.

Determining the antigen targets of CPS-induced functional antibodies will help to shed further light on the developmental stage at which the sporozoite/liver-stage is targeted. CSP and LSA-1 antigens have previously been demonstrated to be predominantly recognized by CPS-induced antibodies, as shown by microarray analysis of 809 *P. falciparum* antigens [48] and enzyme-linked immunosorbent assay and ELISpot analysis (Nahrendorf and Scholzen *et al*., manuscript in preparation). RTS,S vaccination has been shown to elicit antibody titers against only CSP and the highest anti-CSP antibody titers were found in protected volunteers [18,20,21,25]. Other studies, however, found that antibody responses to both CSP and TRAP did not differ between protected and unprotected RAS-immunized volunteers, but protected volunteers were able to recognize novel *P. falciparum* antigens [77,78]. Taken together, this suggests that reactivity of antibodies to pre-erythrocytic or cross-stage antigens other than CSP or TRAP may be associated with protective immunity and, therefore, analysis of plasma samples from (partially) protected CPS-immunized volunteers with a microarray containing ~3,000 *P. falciparum* genes is currently underway. This will be essential to identify novel pre-erythrocytic antigens that are associated with functional activity of CPS-induced antibodies and protective immunity.

Importantly, liver-stage infection in liver-chimeric uPA^+/+^-SCID mice was not completely blocked when using 10 mg/ml IgG (which is equivalent to the typical IgG concentration in human plasma). This indicates that in CPS-immunized volunteers, other immune effector mechanisms may contribute to parasite elimination in concert with antibodies. CPS-induced antibodies may not fully prevent parasite infection, but may reduce the liver parasite load to such a extent, that T-cell mediated responses, for instance, might eliminate the residual infected hepatocytes and thus, prevent blood-stage infection, as observed in previous CPS-trials [45,47].

## Conclusions

In conclusion, CPS-induced antibodies have functional inhibitory activity against pre-erythrocytic stages of *P. falciparum* by inhibiting sporozoite traversal *in vitro* and infection of hepatocytes *in vivo*. These findings highlight the importance of functional antibodies in pre-erythrocytic immunity against malaria and are essential to the current understanding of the mechanisms involved in pre-erythrocytic immunity. Open questions based on these findings that will be addressed in future studies, include the specificity of CPS-induced antibodies and whether their functional activity correlates with CPS-induced protection against malaria.

## Abbreviations

CHMI: Controlled human malaria infection; CPS: ChemoProphylaxis and Sporozoites immunization; CSP: Circumsporozoite protein; FBS: Foetal bovine serum; GAP: Genetically attenuated parasites; IQR: Interquartile range; LSA-1: Liver-stage antigen 1; PBS: Phosphate buffered saline; Pf+: *P. falciparum* positive; PFA: Paraformaldehyde; PfGAP: *P. falciparum* genetically attenuated parasites; uPA: Urokinase-type plasminogen activator; RAS: Radiation-attenuated sporozoites; RT: Room temperature; SCID: Severe combined immunodeficiency; TRAP: Thrombospondin-related adhesion protein.

## Competing interests

The authors declare no competing interests.

## Authors’ contributions

MCB, LF, PM, GLR, CCH, AS and RWS conceived and designed the study. MCB and LF carried out experiments. GJvG provided infected mosquitoes and sporozoites. MCB, LF, EMB, CCH, AS, RWS analysed and interpreted the data. MCB, CCH, AS and RWS wrote the manuscript, and EMB, LF, PM and GLR edited the manuscript. All authors read and approved the final manuscript.

## Supplementary Material

Additional file 1 **Replicates from** *** in vitro *** **traversal experiments.** Replicates from traversal experiments conducted with either 1 mg/ml (A) or 10 mg/ml (B) of pre- or post-immunization IgG from three pools of two volunteers (Study 1) or six individual volunteers (Study 2) are shown. Data are expressed as the mean percentage cells traversed ± SD. Black squares and grey circles represent the percentage cells traversed by sporozoites incubated with either pre-immunization or post-immunization IgG, respectively. Filled symbols show volunteers protected against mosquito challenge. Open symbols represent volunteers with unknown protection status regarding mosquito challenge (blood-stage challenged). Click here for file

Additional file 2 ***In vivo *** **mosquito bite challenge experiments in human liver-chimeric mice.** Data from human liver-chimeric mouse experiments conducted with 10 mg of pre- or post-immunization IgG are shown. Mice injected with PBS could not be directly included in the IgG experiments due to limited availability of chimeric mouse numbers, however, PBS-injected mice used in other parallel experiments performed in the same time frame showed the same range in liver parasite burden as mice receiving pre-immunization IgG. Data are expressed as the median parasite load per million human hepatocytes ± interquartile range. Click here for file

Additional file 3 **Sporozoites incubated with or without 10 µg/ml anti-CSP antibody.** Data from 4 independent traversal experiments conducted with or without 10 μg/ml of monoclonal anti-CSP antibody are shown. (A) Data are expressed as the mean percentage cells traversed. Black circles and squares represent the percentage cells traversed by sporozoites incubated without or with 10 μg/ml anti-CSP antibody, respectively. (B) The percentage inhibition of traversal was calculated for sporozoites only compared to sporozoites incubated with 10 μg/ml anti-CSP antibody. Data are expressed as the median percentage traversal inhibition ± interquartile range. Click here for file
