# Supplementary material for: Sporozoite immunization of human volunteers under chemoprophylaxis induces functional antibodies against pre-erythrocytic stages of Plasmodium falciparum
Source: Malar J. 2014 Apr 5;13:136. doi: 10.1186/1475-2875-13-136 (PMC4113136; doi:10.1186/1475-2875-13-136)
Supplement: Additional file 1 — Replicates from in vitro traversal experiments. Replicates from traversal experiments conducted with either 1 mg/ml (A) or 10 mg/ml (B) of pre- or post-immunization IgG from three pools of two volunteers (Study 1) or six individual volunteers (Study 2) are shown. Data are expressed as the mean percentage cells traversed ± SD. Black squares and grey circles represent the percentage cells traversed by sporozoites incubated with either pre-immunization or post-immunization IgG, respectively. Filled symbols show volunteers protected against mosquito challenge. Open symbols represent volunteers with unknown protection status regarding mosquito challenge (blood-stage challenged). [file 1475-2875-13-136-S1.pdf]

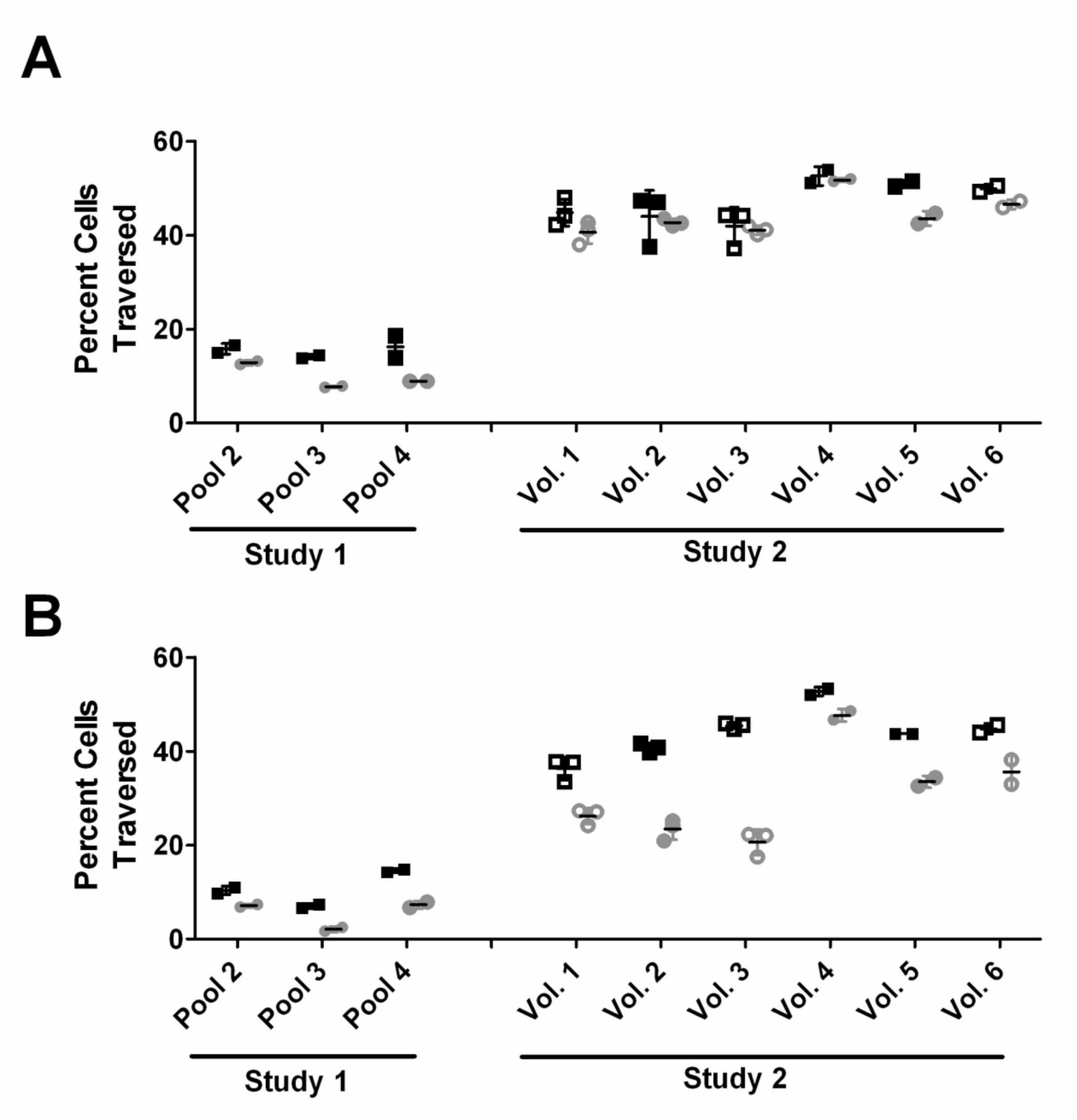

Supplementary Figure 1. Replicates from *in vitro* traversal experiments.

Replicates from traversal experiments conducted with either 1 mg/ml **(A)** or 10 mg/ml **(B)** of pre- or post-immunization IgG from three pools of two volunteers (Study 1) or six individual volunteers (Study 2) are shown. Data are expressed as the mean percentage cells traversed ± SD. Black squares and grey circles represent the percentage cells traversed by sporozoites incubated with either pre-immunization or post-immunization IgG, respectively. Filled symbols show volunteers protected against mosquito challenge. Open symbols represent

volunteers with unknown protection status regarding mosquito challenge (blood-stage challenged).
