# Supplementary material for: Sporozoite immunization of human volunteers under chemoprophylaxis induces functional antibodies against pre-erythrocytic stages of Plasmodium falciparum
Source: Malar J. 2014 Apr 5;13:136. doi: 10.1186/1475-2875-13-136 (PMC4113136; doi:10.1186/1475-2875-13-136)
Supplement: Additional file 2 — In vivo mosquito bite challenge experiments in human liver-chimeric mice. Data from human liver-chimeric mouse experiments conducted with 10 mg of pre- or post-immunization IgG are shown. Mice injected with PBS could not be directly included in the IgG experiments due to limited availability of chimeric mouse numbers, however, PBS-injected mice used in other parallel experiments performed in the same time frame showed the same range in liver parasite burden as mice receiving pre-immunization IgG. Data are expressed as the median parasite load per million human hepatocytes ± interquartile range. [file 1475-2875-13-136-S2.pdf]

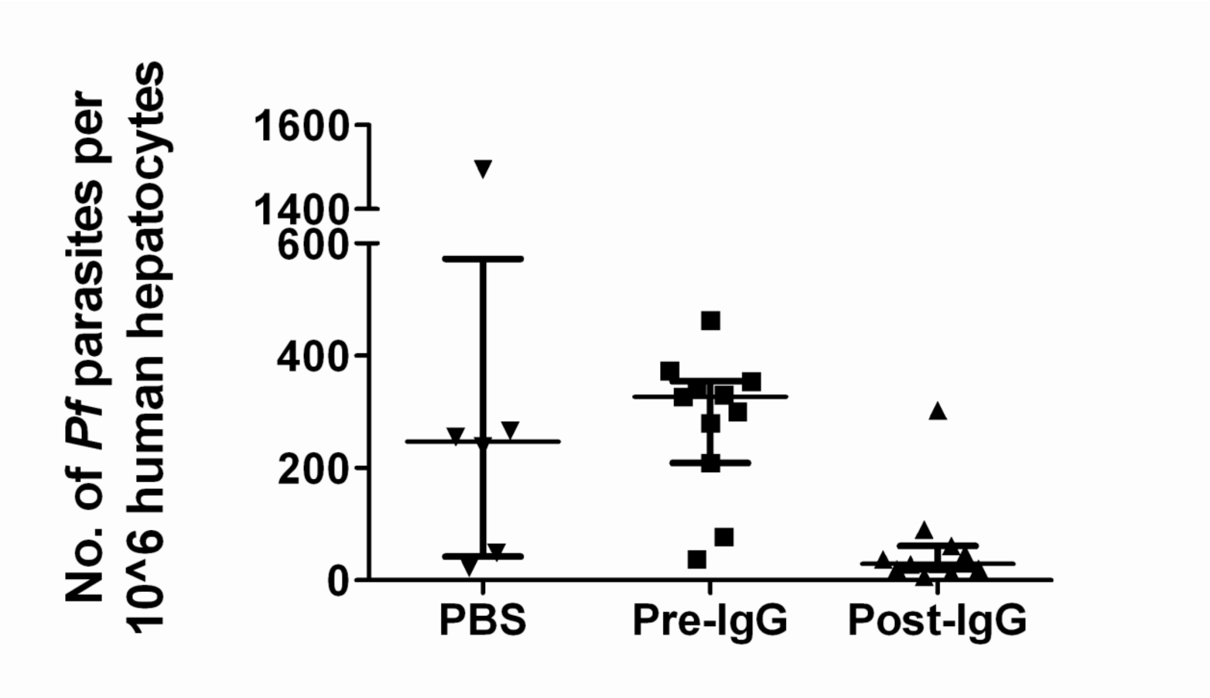

Supplementary Figure 2. ***In vivo*** **mosquito bite challenge experiments in human liver-chimeric mice.**

Data from human liver-chimeric mouse experiments conducted with 10 mg of pre- or post-immunization IgG are shown. Mice injected with PBS could not be directly included in the IgG experiments due to limited availability of chimeric mouse numbers, however, PBS-injected mice used in other parallel experiments performed in the same time frame showed the same range in liver parasite burden as mice receiving pre-immunization IgG. Data are expressed as the median parasite load per million human hepatocytes ± interquartile range.
