# Supplementary material for: Sporozoite immunization of human volunteers under chemoprophylaxis induces functional antibodies against pre-erythrocytic stages of Plasmodium falciparum
Source: Malar J. 2014 Apr 5;13:136. doi: 10.1186/1475-2875-13-136 (PMC4113136; doi:10.1186/1475-2875-13-136)
Supplement: Additional file 3 — Sporozoites incubated with or without 10 µg/ml anti-CSP antibody. Data from 4 independent traversal experiments conducted with or without 10 μg/ml of monoclonal anti-CSP antibody are shown. (A) Data are expressed as the mean percentage cells traversed. Black circles and squares represent the percentage cells traversed by sporozoites incubated without or with 10 μg/ml anti-CSP antibody, respectively. (B) The percentage inhibition of traversal was calculated for sporozoites only compared to sporozoites incubated with 10 μg/ml anti-CSP antibody. Data are expressed as the median percentage traversal inhibition ± interquartile range. [file 1475-2875-13-136-S3.pdf]

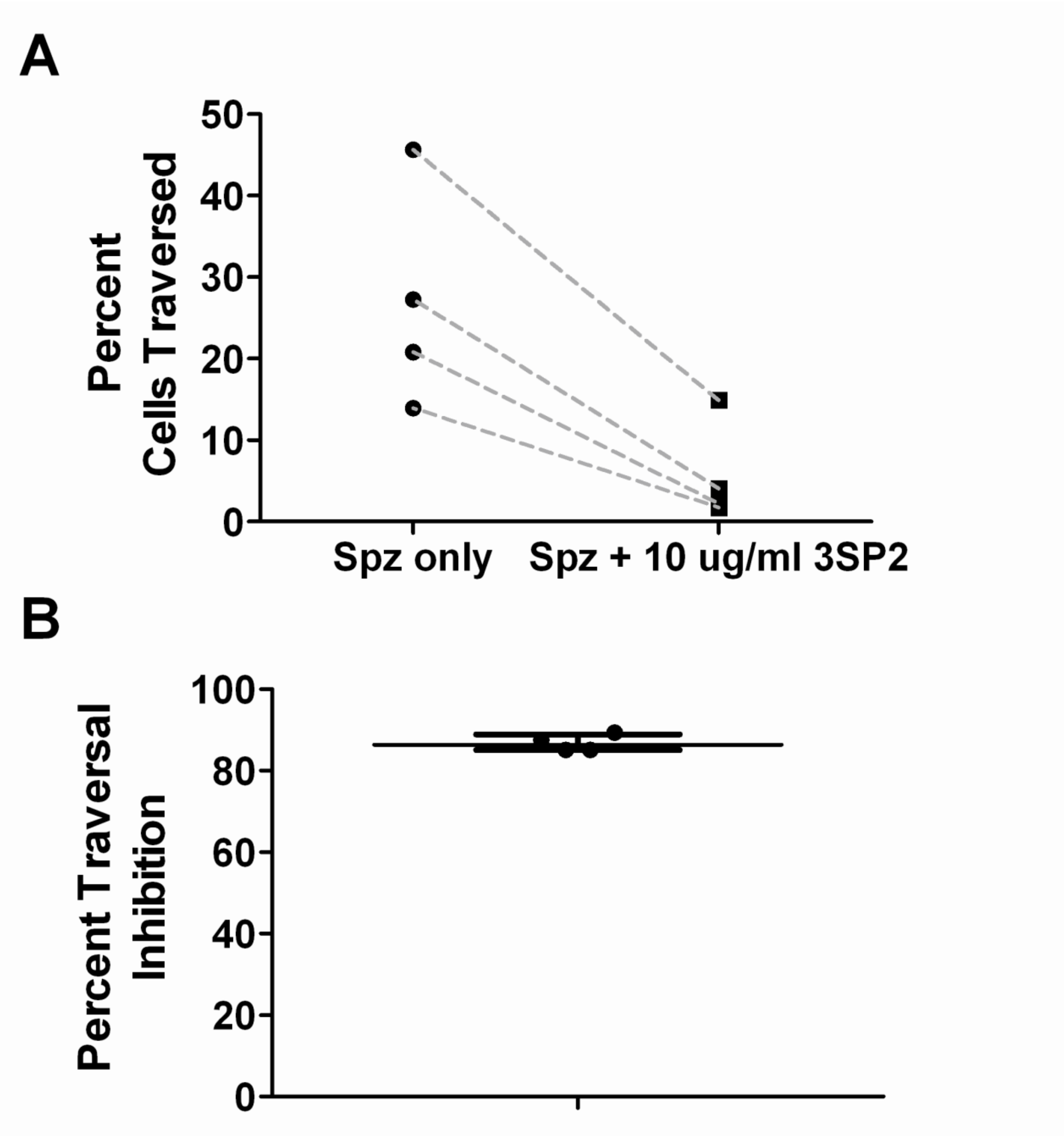

Supplementary Figure 3. Sporozoites incubated with or without 10 µg/ml anti-CSP antibody.

Data from 4 independent traversal experiments conducted with or without 10 µg/ml of monoclonal anti-CSP antibody are shown. **(A)** Data are expressed as the mean percentage cells traversed. Black circles and squares represent the percentage cells traversed by sporozoites incubated without or with 10 µg/ml anti-CSP antibody, respectively. **(B)** The percentage inhibition of traversal was calculated for sporozoites only compared to sporozoites

incubated with 10 µg/ml anti-CSP antibody. Data are expressed as the median percentage traversal inhibition ± interquartile range.
